# Letter from Dr. C. H. Hughes

**Published:** 1906-10

**Authors:** C. H. Hughes


### Correspondence.

### Letter From Dr. C. H. Hughes.

### THE WORK OF THE CONGRESS ON TUBERCULOSIS.

*Dr. F. E. Daniel, President American International Congress on Tuberculosis; Editor Texas Medical Journal, Austin, Texas.*

MY DEAR DOCTOR DANIEL: While tuberculosis is not in my special line of work, it is related to it as a micro-toxic sequence of nervous break-down, exhaustion and diminished organic resistance, and I am interested in all movements that tend to popularize and disseminate the views of the medical profession to the necessity of preventive sanitary vigilance regarding invasion and spread among the people. Every movement that keeps the alarm sounded and points to roads and means of safety, and tends to secure active measures and efficient methods should have professional countenance, and the more popular and general the alarm and the sanitary resistence to this silent scourge, the better for our common humanity and the glory of that great profession which points the way to safety and offers means of rescue.

Right knowledge is the beginning of wisdom on this subject, but the light has been shining from the profession on the people in the darkness for lo! these many years, and the darkness has, till of late, comprehended not.

The increase of popular comprehension of the insidious spread and dangers of tuberculosis, through medical effort and work like yours, now assures the dawn of safety at some time not far distant from the enormous fatalities from the on-marching great white plague, assuring ultimate redemption from the threatened destruction.

The people now know that the promiscuous tuberculous spitter in public places may kill with his deadly saliva; they know that food and drink and second-hand clothing may spread it; that glasses at bars and soda fountains, dipped in water not often renewed, may spread it. That dairies may be as dealy as the apothecary's poison; and likewise the butchers' stalls and the beef packing places; that the dust of the street may carry the seeds of death, and edibles, exposed for sale, unprotected from dust, may kill while they nourish. But enough of the people do not yet know or rightly ponder these fatal facts, to demand safety. The ice man still drags his ice blocks over the filthy pavements; putrifying garbage is still exposed in the alleys or carted through the streets, and the street car hog expectorates disease upon the floors; but publicity of the dangers of tubercular infection will spread through efforts like yours, as publicity of political crime has spread in Missouri, till popular knowledge and demand for remedy will secure the people's rescue.

The people will stop the breeding of tuberculosis, as they will that of the death-bearing mosquitoes, and the perpetrators of political crime. Popular sanitation is in the air, and the germs of disease and political degeneration and money getting and moral prostitution are destined to die in this fair land of ours, from the same cause, viz.: an aroused determination to bring about states of physical, mental, moral and political health, that the people in this fair heritage of our great and good fathers may live out a worthy and useful destiny.

Line upon line and precept upon precept have been presented to the people and many tuberculosis experts by our profession free of charge and illustrations of the fatality of neglect of our warnings may be found in the sad experience of nearly every family in the land, yet the people are not sufficiently active in exterminating this plague of the twentieth century.

I could not help noting how comparatively few of the population and visitors were to be seen at the free tuberculosis exhibit at Toronto during the late meeting of the British Medical Association, notwithstanding the prominently posted assurance of the fact that there was no danger, as there is none, of course, from such exhibitions of the bacillian cause and ravages of this terrible disease under the ordinary antiseptic precautions which protect physicians as well as others from contagion.

If you can move the imperiled people one step further in the direction of safety, and induce our lawmakers to take more interest in this phase of sanitary science upon which you are aiming to enlighten and stimulate to protective and defensive action, your work will not have been in vain. Popular sanitary congresses, under right scientific medical guidance ought to be encouraged, after the medical profession has so clearly outlined the danger and the ways of safety as it has in regard to tuberculosis.

The next thing for the people and the profession to jointly take up for popular sanitary security is the mosquito and the stoppage of his deadly work, as was done lately at New Orleans and before that, and first, by our profession in Havana, as it is now being done through the medical department of the government on the Panama Canal belt.

When the recommendations of the profession as to these death-dealing scourges shall have been in practice by governing authority, and personal, popular precautions, it will be recalled then how much more than armies to the public weal are the recommendations of wise physicians and expert bacteriologists if rigorously carried out in practical measures of rescue.

An era of popular sanitary warfare is upon us. The army of disease extermination is enlarging by volunteers joining the regulars in medical science, and the fruits of victory are coming in the turning of politicians into sanitary statesmen, to help save the people in their health and lives. The fruition of the true medical man's hopes, and the reward of his living, silent, patient labor, is in sight, through widespread popular knowledge and combined effort with that of the medical profession; and when the final triumph comes, let it not be forgotten that the enlightened, resourceful, silently-working medicine has pioneered the people out from the disease perils that walked in darkness and beset them with evils they had not seen but for the light of our profession freely given. When the rescue comes, let it be proclaimed and inscribed: "Through the light of medical science the plague was stayed"; for, too often the people receive the benefactions of our great science and art, yet forget their benefactors and the source of their safety, turning to popular fakes and fads for relief that fails.

After the tubercle bacillus, the anophile and the stegomyia shall have been effectually quarantined and destroyed by State action, may it not be hoped that municipal attention may be turned to the prevention of the spread of neuropatic and psychopathic degeneracy among our people, instead of furnishing only those cemeteries for brain-damaged imbeciles, idiots, epileptic, chronic insane and paralysis which, though the best past professional knowledge and experience could devise and command, are better memorials to our philanthropy than to our knowledge of the prevention of psychoneural decadence.

The active interest of our national government, expressed through Secretary Root's efforts and the interest of his predecessor, Secretary Shaw, in the previous Congress, are hopeful signs of coming triumph over this scourge of humanity on this continent. It is to be hoped that active government interest will continue to be manifested toward yours and all other contemporaneous efforts for tuberculosis eradication and mosquito extermination.

Sanitary problems are subjects for the highest statesmanship and paramount government interest and endeavor.

Very truly yours,

C. H. Hughes,

[Dr. Hughes is the distinguished editor of *The Alienist and Neurologist,* and late professor of nervous diseases in Marion-Sims Medical College. He is also honorary president of the American International Congress on Tuberculosis, which will meet in New York City in November to discuss the problem of tuberculosis prophylaxis.—Ed.]

